# Domain and Dendrite Pattern Formation in PbSe Nanocrystal Monolayers

**DOI:** 10.1021/acs.cgd.6c00339

**Published:** 2026-08-03

**Authors:** Paolo Accordini, Willem J. P. van Enckevort, Elias Vlieg

**Affiliations:** 6029 Radboud University, Institute for Molecules and Materials, Heyendaalseweg 135, 6525 AJ Nijmegen, The Netherlands

## Abstract

PbSe nanocrystal monolayers are deposited on ethylene glycol substrates as a function of nanocrystal surface concentration. Two different structural domains were encountered: low density (LD) domains composed of a close packing of nonattached crystals and high density (HD) domains made up of strings of oriented attached nanocrystals. For the lowest surface concentrations only LD layers form; at higher concentrations HD domains develop in the LD phase. Interestingly, at even higher concentrations LD seaweed type dendrites develop by diffusion transport in an extended and rapidly formed HD layer, densely populated with nonattached nanocrystals. The driving force for this remarkable dendrite growth is the rearrangement of the HD layers by mutual alignment, expelling nonbonded nanocrystals from these layers.

## Graphical abstract

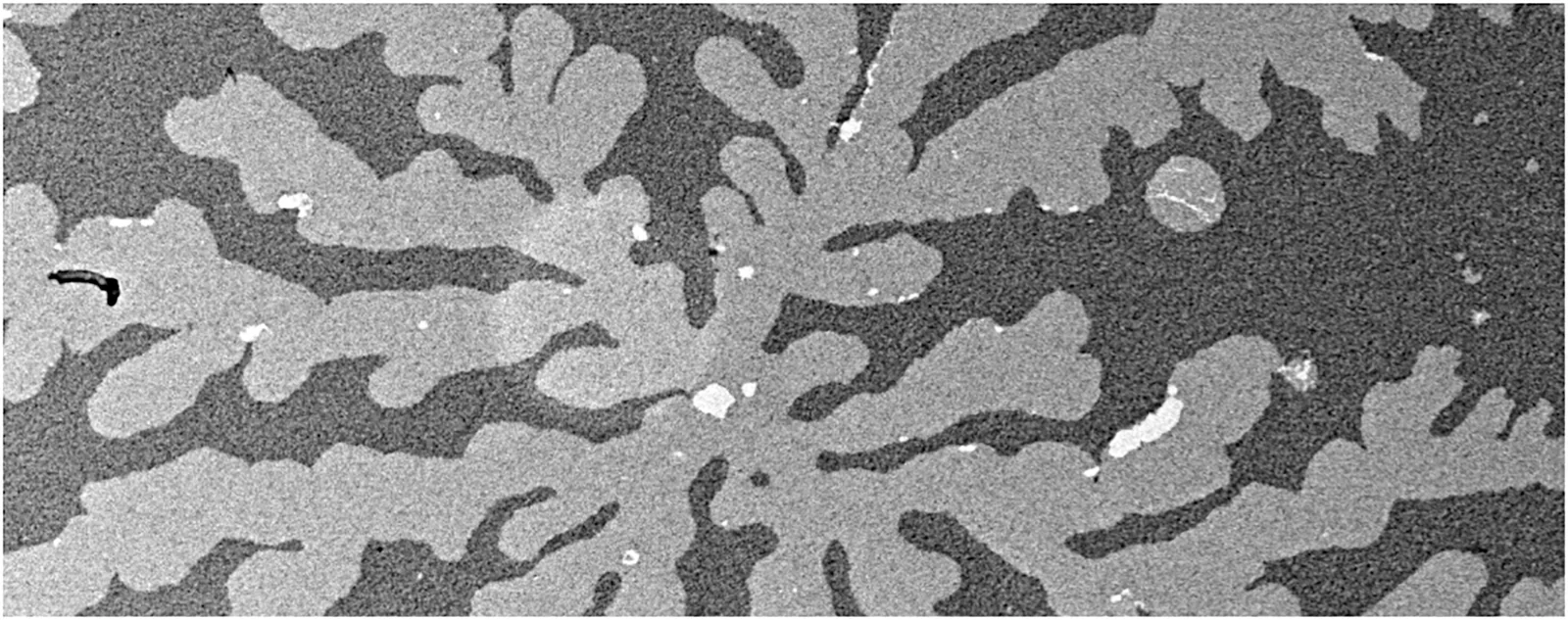

## 1 Introduction

Lead-chalcogenide nanocrystals, in particular those composed of lead-sulfide (PbS) and lead-selenide (PbSe), have extensively been studied for their abilities to form monolayers exhibiting long-range ordering and coherent fusion between adjacent units.^1–3^ Both materials are able to form square or hexagonal lattices, the latter being the most desired form owing to its possible electronic properties.^4,5^ Also less ordered monolayers have been reported, which consist of an assembly of isolated strings of interconnected nanocrystals.^6,7^ Many works in the literature have already explored experimental routes to the synthesis of these materials.^8^ However, no reliable protocol to selectively synthesize a specific monolayer type has been found yet.

All these experiments are performed using the so-called drop cast method in which a nanocrystal solution is drop casted on top of a substrate of immiscible liquid. Then, the system is allowed to reach equilibrium, after which the liquid solvent evaporates under controlled conditions. During this step nanocrystals are brought to the liquid–liquid interface, where they get adsorbed forming a monolayer.^9^ The amount of nanoparticles and the properties of the interface dictate how the system evolves from chaos to a completely or partially ordered monolayer.^10^

During our investigation of the PbSe monolayer properties as a function of nanoparticle surface density, aside from domain structures, we unexpectedly observed the formation of extensive dendritic patterns within a specific range of nanocrystal concentrations. Figure 1 presents examples of such a dendritic structures observed through electron microscopy. The dendrites are composed of a low-density (LD) phase, formed in a high-density (HD) phase.

**Figure 1:**
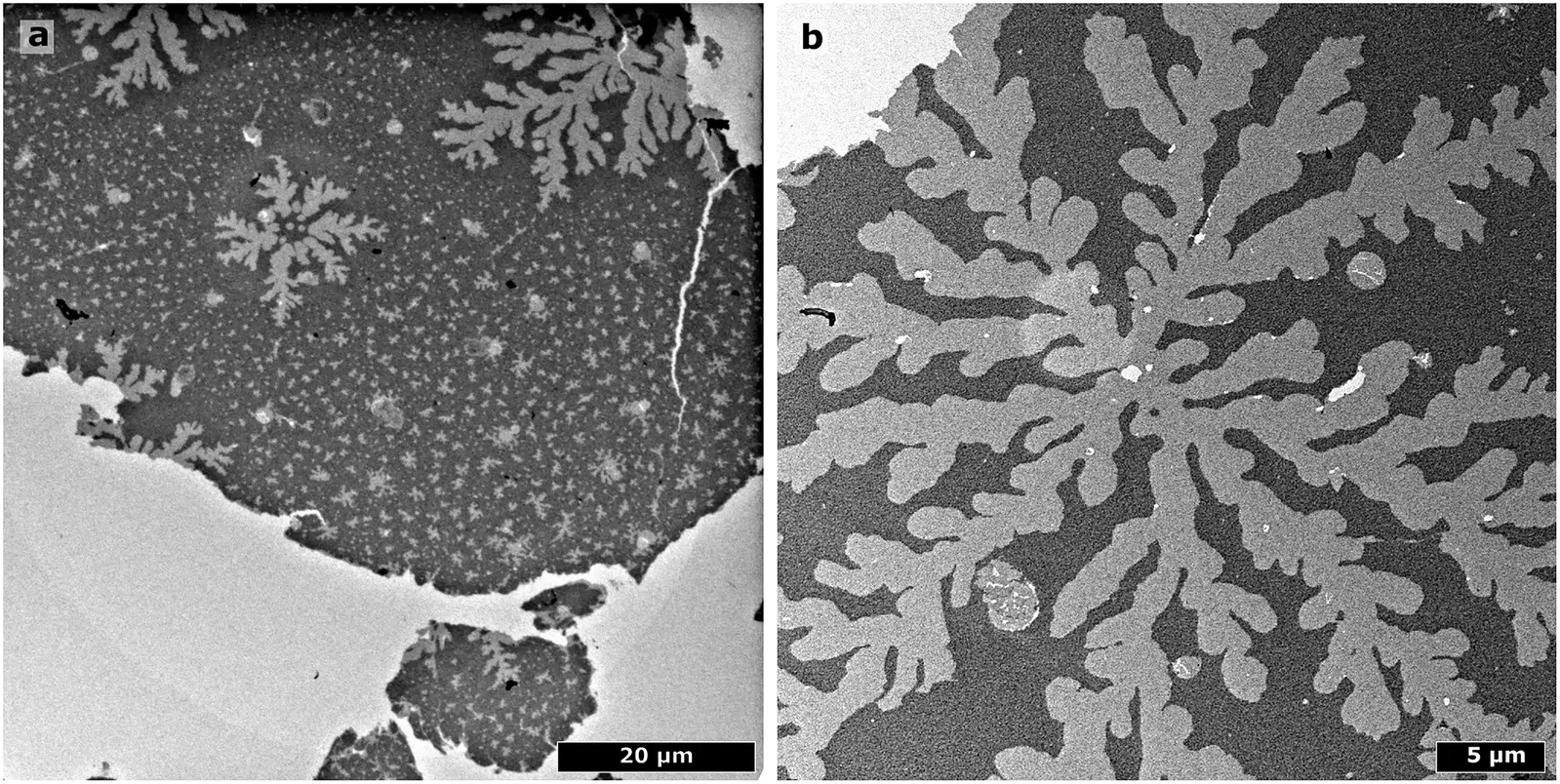
TEM images of dendritic growth patterns found in monolayer samples prepared using higher nanocrystal concentrations. (a, b) are two representative samples taken at a different magnification.

Dendrite growth is a common and well studied phenomenon in the field of crystal growth.^11,12^ These branched structures are formed if the growth of a solid from a fluid phase is controlled by mass or heat transport, for instance by diffusion of growth units in a solution toward the crystal surface. The formation of these patterns is strongly enhanced by a high driving force (supersaturation) and a low liquid–solid interfacial energy. The shape of these patterns is largely controlled by the anisotropy of the interfacial energy of the two phases.^13^

To gather insight in the formation process of the unexpected dendrite features in the monolayers, we studied the structural characteristics of the two phases involved and the role of the nanocrystal concentration. The LD phase is composed of nonattached nanocrystals and behaves as a partially ordered liquid of high viscosity. The HD phase is a compacted assembly of strings of attached nanocrystals having solid-like properties. Our results show that dendritic patterns emerge only within a specific range of higher nanocrystal surface densities. Surprisingly, in contrast to the classical dendrite growth models, we observed that the dendrites are composed of the liquid-like LD phase instead of the HD phase. The formation of domain and dendrite patterns, along with a possible explanation for the above discrepancy, will be addressed in this work.

## 2 Methods

### 2.1 Materials

Lead acetate trihydrate (99.999%, Merck), Selenium (99.999%, Alfa Aesar), Oleic acid (OA, 90% Merck), Octadecene (ODE, 90% Merck), Diphenylphosphine (DPP, 98%, Merck), Trioctylphosphine (TOP, 97% Merck), Toluene (99.8% anhydrous, Merck) and Octane (99.999%, Merck). ODE was thoroughly degassed under vacuum overnight before use. TOP was degassed at 160 °C under vacuum overnight and stored in a glovebox. All other chemicals were used without any other purification steps.

### 2.2 Nanocrystal Synthesis

PbSe nanocrystals used in this study were prepared following a method adapted from the synthesis route described by Steckel et al.^14^ Synthesis was performed in a Schlenk line using standard oxygen free techniques. For the lead oleate precursor: 11.38 g of lead acetate trihydrate was mixed with 25.46 g OA and 37.91 g ODE in a three-necked round-bottom flask. This vessel was connected to the Schlenk line, its atmosphere was cycled with nitrogen and vacuum three times and heated up to 80 °C under nitrogen atmosphere. Vacuum was applied when bubbling subsided and it was left degassing overnight to remove any trace of acetic acid. The trioctylphosphine selenide (TOPSe) precursor was prepared inside a water and oxygen free glovebox (<1 ppm of O_2_ and <1 ppm of H_2_O in N_2_) by dissolving 2.52 g selenium in 16 mL of TOP.

For the synthesis of nanocrystals: inside the oxygen-free glovebox, a three-necked flask was loaded with a stirring bar, 4.64 g of the lead precursor and 560 mg OA. ODE was added until reaching 10 mL total volume. A sealable syringe was prepared with a mixture of 8 mL TOPSe, 139 μL DPP and 2 mL ODE. The three-necked flask was connected to the Schlenk line and its atmosphere was cycled three times. Then, the solution was heated up to 180 °C and the selenium precursor was swiftly injected into the flask. Subsequently the temperature was kept constant at 130 °C for 90 s, after which 20 mL butanol was injected to quench the reaction. The vessel was allowed to cool down to room temperature before proceeding. Finally, 10 mL of methanol was added to force the precipitation of the quantum dots and the mixture was centrifuged at 2500 rpm for 10 min. Supernatant was discarded and the solid pellet was redispersed in fresh anhydrous toluene. This washing procedure was repeated 3 times before storing the nanocrystals in toluene as a stock solution with a concentration of 3.3 × 10^–5^ μg/μL in the glovebox for long-term usage.

### 2.3 Nanocrystal Characterization by Infrared Spectroscopy

The size of the nanocrystals was determined by infrared spectroscopy, using the model developed by Moreels et al.^15^ For this the samples were prepared in the glovebox by drying under vacuum 25 μL of stock solution and redispersing the quantum dots in tetrachloroethylene (TCE). Samples were transferred into a sealed quartz cuvette and analyzed using a Bruker Vertex 80 spectrophotometer. We found that the nanocrystals used in this work were 5.65 nm in diameter, excluding the ligands.

### 2.4 Growth of Monolayer Samples

Experiments are performed in the glovebox. A Petri dish (diameter 30 mm) partly filled with 7 mL ethylene glycol (EG) is used as a substrate. The amount of stock solution needed for each experiment is calculated by assuming partial or total surface coverage. Usually 4 to 9 μL of the stock solution mentioned above is dried in vacuum and then redispersed in 35 μL octane. Then, the 35 μL nanocrystal suspension in octane is drop cast on the ethylene glycol forming a thin suspension layer. To avoid rapid solvent evaporation the Petri dish is placed in a larger Petri dish with a reservoir of octane. In addition, the whole set up is covered by an upside down placed beaker with a small opening. In combination with the extra solvent, the atmosphere inside the enclosed volume is in quasi-equilibrium, thus slowing down the evaporation process. The temperature is about 20 °C. Under the conditions described here, 35 μL of octane takes about 20–30 min to completely evaporate. The monolayer and subsequent dendrite formation occurs at the interface of ethylene glycol with air and, initially, octane, see e.g.,^9^ for in situ observations. After 3 h, the thin film was scooped using a TEM grid (copper/carbon film, 200 mesh) and left under vacuum overnight to remove excess ethylene glycol. We found that waiting much longer than 3 h often resulted in destroyed patterns, because of the continued stripping of the ligands from the nanoparticles. In our experiments one monolayer of nonattached nanocrystals corresponds to ≈6 μL stock solution (after drying) in the 35 μL suspension added to the system. This corresponds to 1.4 × 10^4^ nanocrystals/μm^2^ when the nanocrystals are not attached. In order to distinguish the observed behavior from occasional issues in these difficult experiments, each experiment was repeated three times and only the regular behavior is reported here. Figure 2 shows a schematic of the experimental setup as well as a typical set of samples under the conditions of dendrite formation.

**Figure 2:**
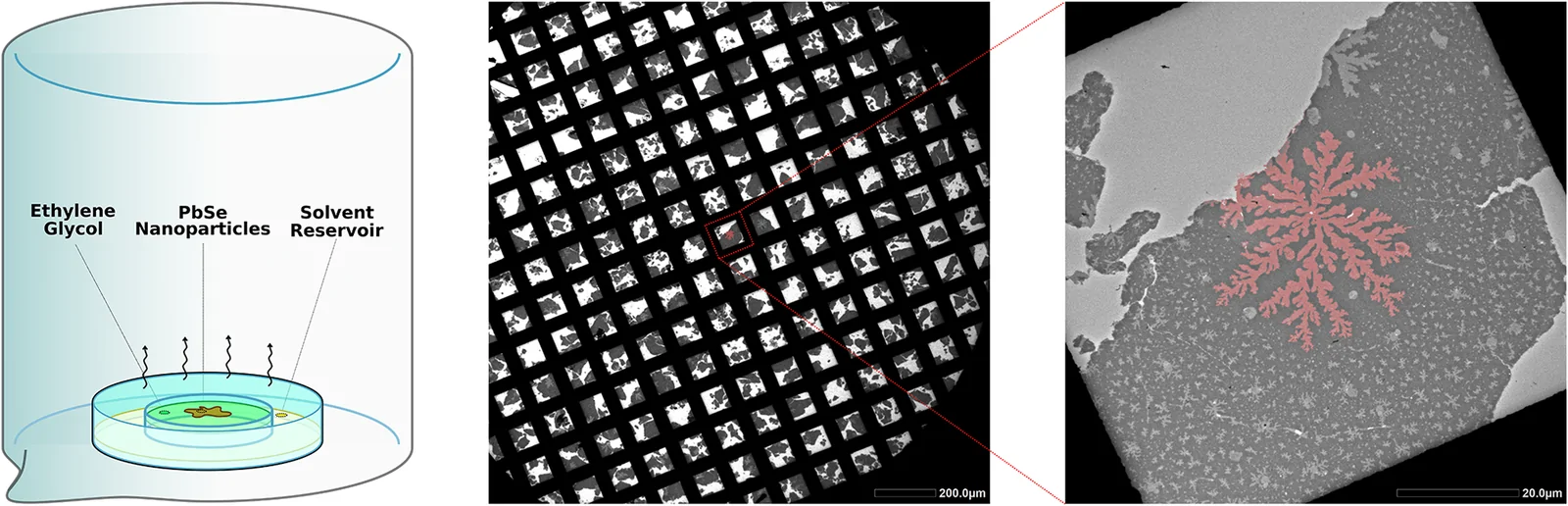
Left: a schematic of the experimental setup. A beaker is placed on top of our system, composed of the ethylene glycol substrate and an extra reservoir of solvent. The reservoir is used to saturate the atmosphere of the beaker with solvent vapors, thus slowing down the evaporation process. The middle and left figures are TEM pictures of one sample taken at different resolutions. It is possible to observe the dendritic patterns randomly distributed over the surface of the sample, which shows that the dendritic growth is not a localized phenomena.

### 2.5 Characterization by TEM

Transmission electron microscopic images were obtained using a JEOL TEM 1400 operating at 120 keV. The exposure time was kept at 500 ms for every image shown in this work. Samples on the TEM grid were prepared as described in the previous section and used without any further modification. The ImageJ software package was used to enhance contrast and perform measurements on the images. Careful TEM measurement of nonattached nanocrystals in highly ordered LD domains gave a ligand embedded nanocrystal diameter of 8.6 nm, corresponding to 1.4 × 10^4^ nanocrystals/μm^2^ if in perfect hexagonal setting.

## 3 Results

### 3.1 Two Different Phases

In observing the domain and dendrite patterns in the grown PbSe nanocrystal monolayers by TEM, we found two different phases in all cases (except for the 4 μL stock solution). Figure 3 displays the two phases imaged by TEM at different magnifications. As the gray scale of the micrographs scales with electron scattering intensity and thus with nanocrystal density, the half bright domains (red overlay) correspond with a phase of lower nanocrystal density (LD) and the darker parts (green overlay) with a high density (HD) phase.

**Figure 3:**
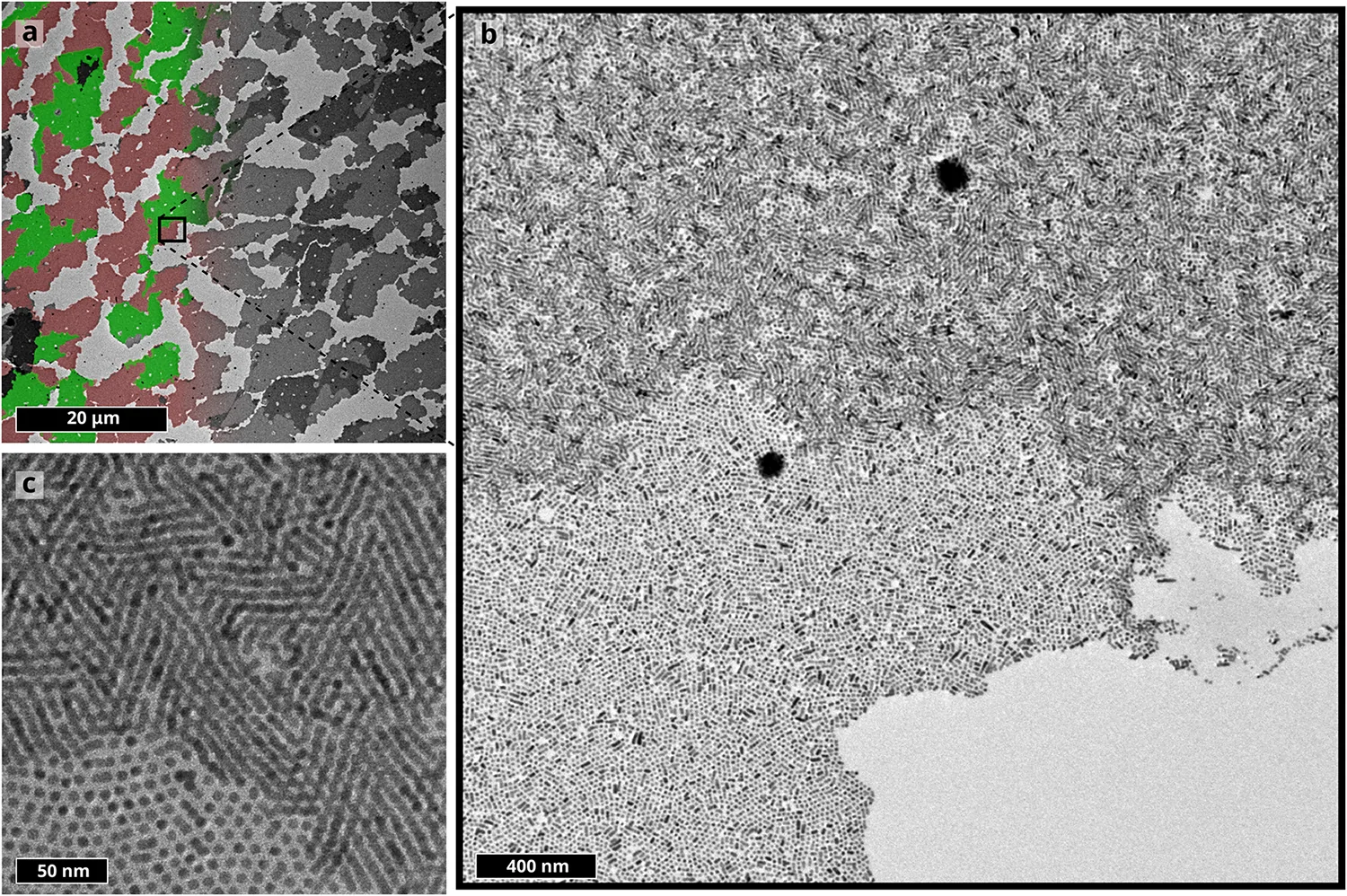
(a, b) TEM images recorded at different magnifications showing the different regions in a monolayer PbSe nanocrystal film obtained after the evaporation of a 5 μL suspension. Red overlay is used to highlight LD regions, while green represents the HD phase. (c) Details of the boundary between LD and HD.

At high nanocrystal concentrations dendrites are formed with a large number of branches. The half bright dendrite pattern in Figure 1 indicates that this branched feature is composed of LD phase, developed in a matrix of HD phase. The very bright parts in Figures 1 and 3b are empty spaces formed by rupture of the LD and HD domains during separation of the nanocrystal monolayer film from the EG substrate prior to electron microscopy. To solve the riddle of the surprising dendrite growth in our PbSe monolayers, first a detailed characterization of the two phases is needed.

Closer examination of the specimens reveals that the LD domains are composed of contacting, nonattached nanocrystals (Figure 4a). The number density of the nanocrystals is 1.25 ± 0.02 × 10^4^ nanocrystals/μm^2^, so the average distance is 9.6 nm, which is slightly larger than the nanocrystal size of 8.6 nm including the ligands.

**Figure 4:**
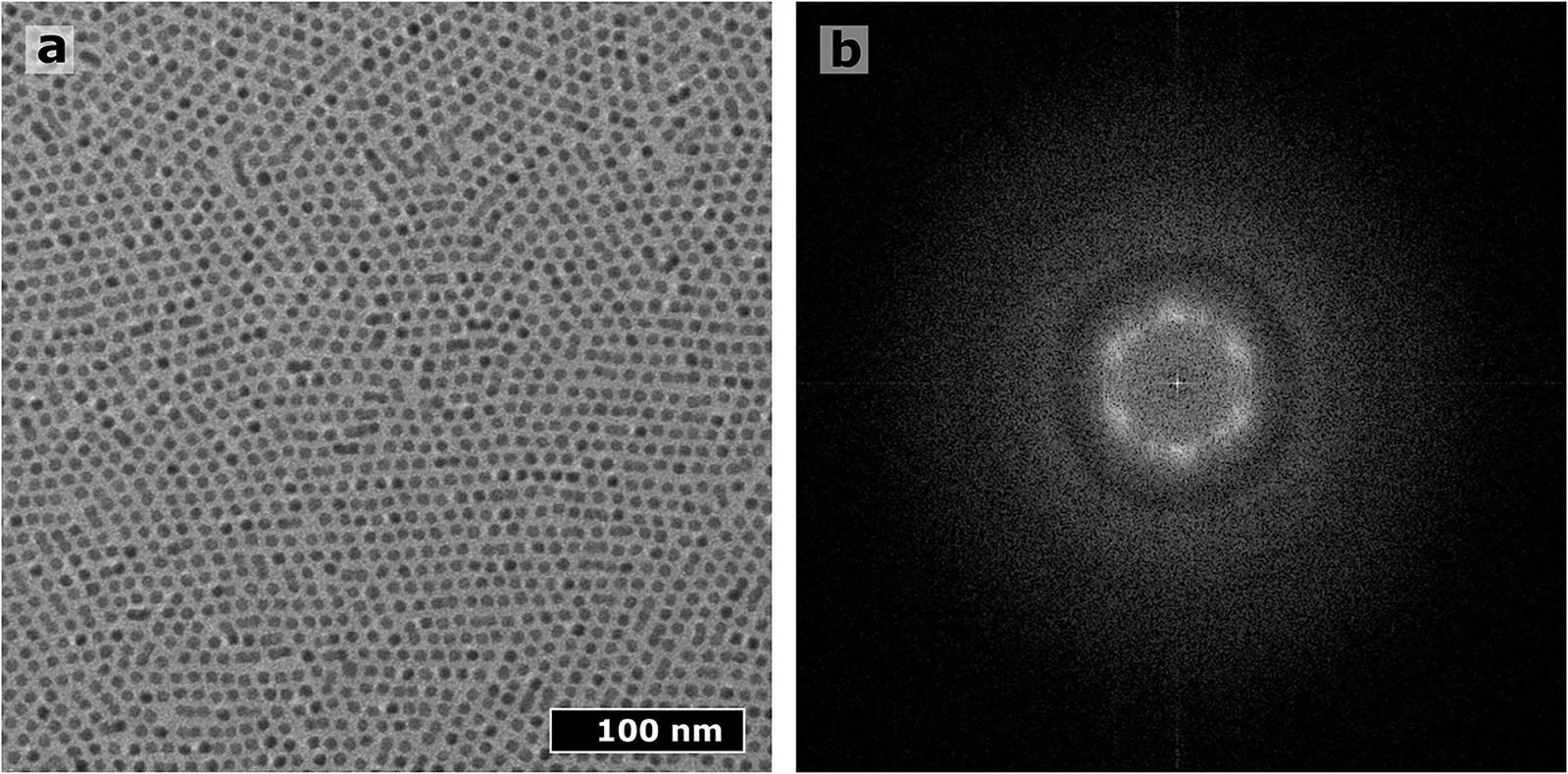
LD domain composed of nonattached PbSe nanocrystals, which are partially ordered in a hexagonal close packing (8.5 μL stock solution): (a) High magnification TEM image; (b) Fourier transform.

Given this core–core distance, it follows that ligands are still present. The liquid-like patterns show some hexagonal ordering, induced by close packing of the nanocrystals (Figure 4a). This can be seen from the Fourier transform in Figure 4b. This points to some mobility of the units in the layer, allowing reorganization of individual units in the layer.

The HD domains comprise linear chains of attached nanocrystals with a number of nonattached specimens in between (Figures 3c and 5a). A double layer of close packed nonattached nanocrystals can in principle explain some of of the HD-layer properties, but is unlikely to occur as elucidated in the Supporting Information. The nanocrystal density is 2.7 ± 0.02 × 10^4^ nanocrystals/μm^2^, which is considerably higher than that of the LD domains. Fourier transformation of the HD TEM image agains reveals hexagonal ordering (Figure 5b). The linear strings in the HD domains are composed of so-called zigzag chains in which the NaCl type PbSe nanocrystals attach with their {1 0 0} facets in a buckled fashion.^6^ The distance between adjacent nanocrystals in the strings is 4.6 ± 0.1 nm, determined by counting the number of nanocrystals in a string of given length. These strings often show a turn off by an angle of 120 ± 8° (Figures 3c, 5, and 6b). These angles point to an averaged trigonal/hexagonal symmetry and indicate that the [1 1 1] directions of the zigzag strung nanocrystals are perpendicular to the substrate.

**Figure 5:**
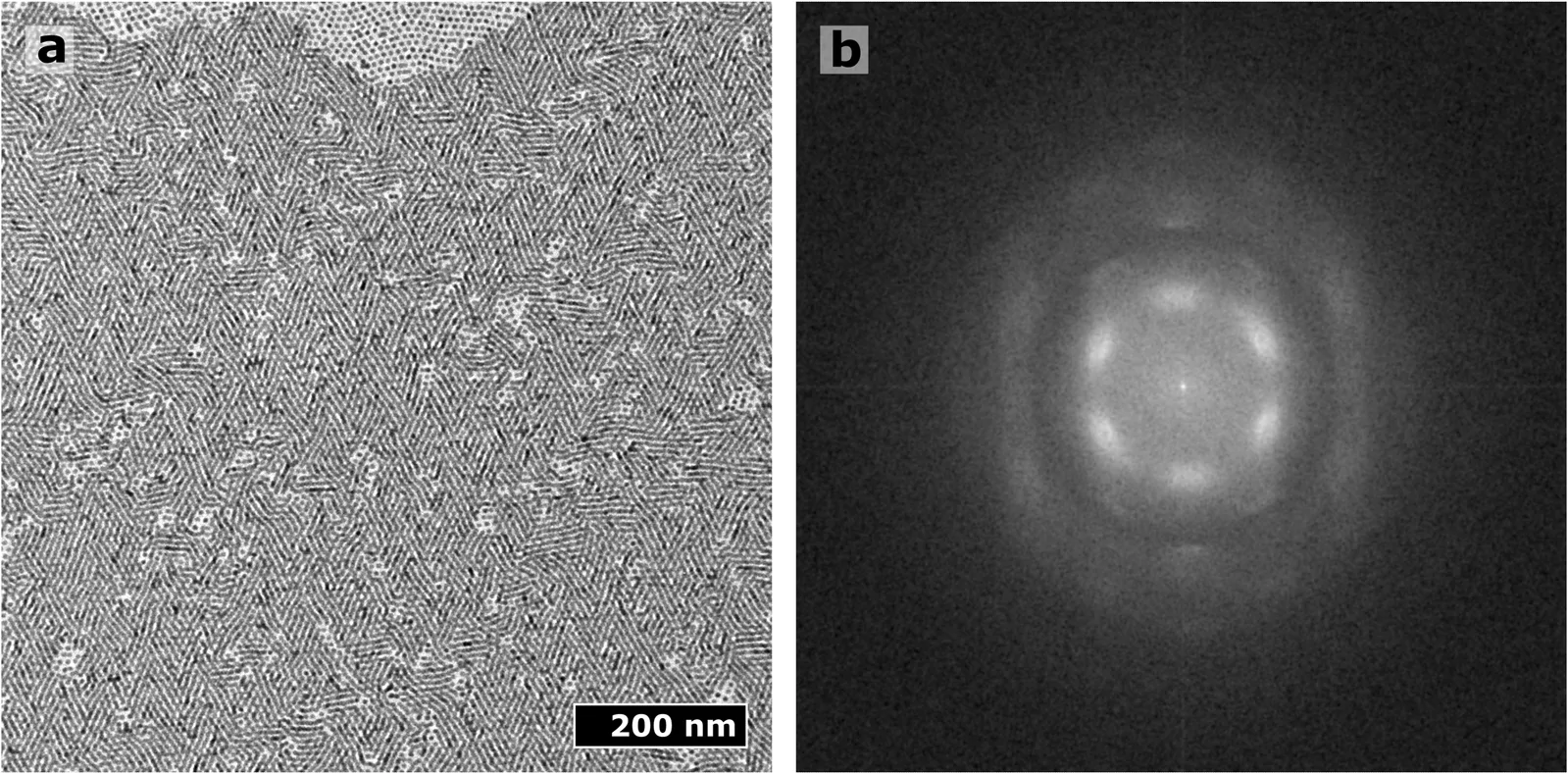
HD domains partially ordered by hexagonal packing of chains made up of zigzag attached nanocrystals (8.5 μL stock solution): (a) TEM image; (b) Fourier transform.

**Figure 6:**
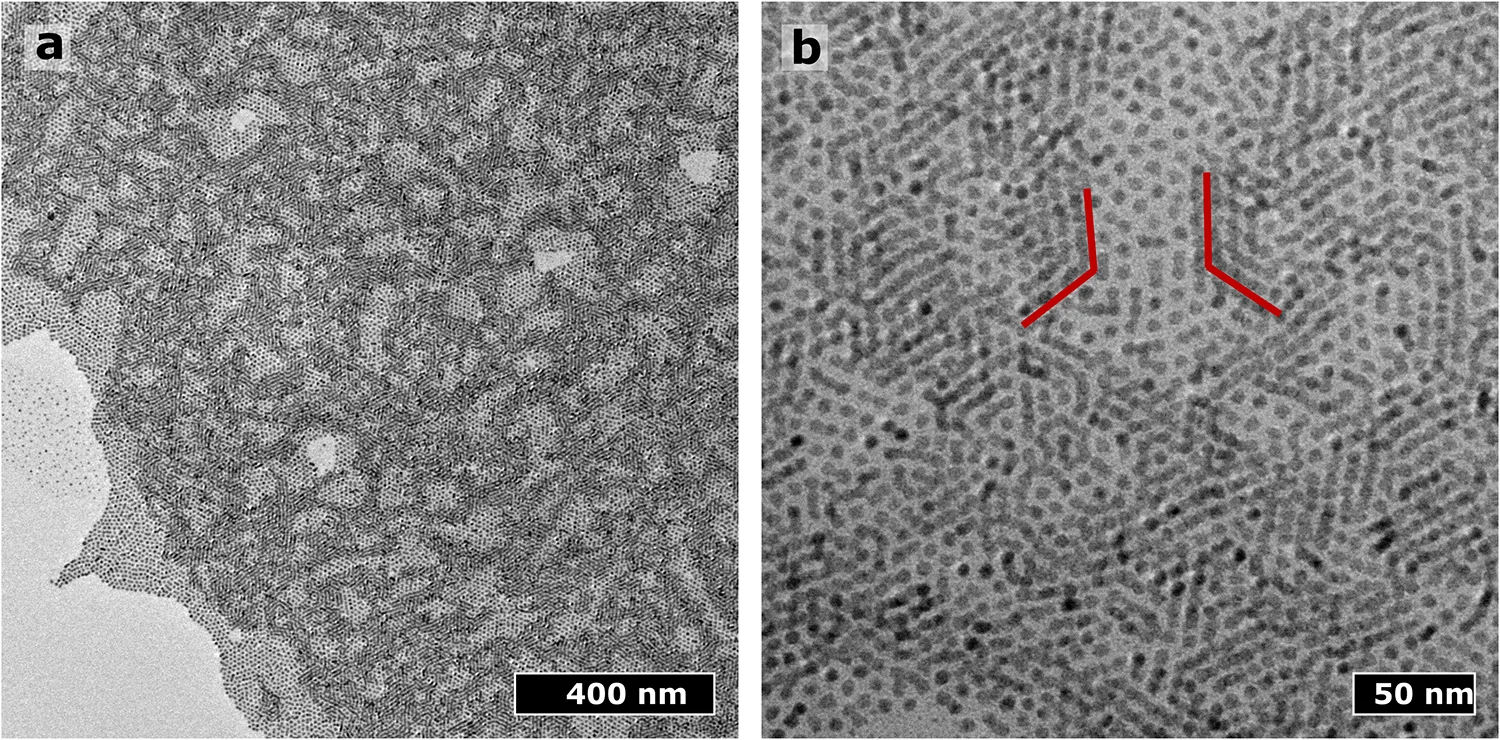
Intermediate state of domain formation showing a mixture of small LD and HD domains (7.5 μL stock solution). In (b), two cases of a 60° bend in the chains are highlighted by the red lines.

It is clear that attachment preferentially occurs straight-on at the end points of the attached chains and in a few cases (1:10–50) sideways attachment takes place at the tips, leading to the observed 120° side branching. Nonattached parallel alignment of the strings commonly happens and leads to the observed (Figure 5b) local hexagonal ordering, which is induced by the 120° turn off angle of many chains.

Parallel strings coherently attached to each other were never found; this follows from the interline spacing of 7.5 nm, which is slightly less than the 8.5 nm separation of nonattached units, but far more than the 4.6 nm spacing of the connected units in the strings. Based on what we have observed in our samples, nanoparticles tend to form prevalently strings in high-density regions: this suggests that the activation barrier for nanocrystal attachment is lower for the end points of the chains when compared to sideways attachment. Chain growth stops after collision with a nonparallel adjacent string, likely because there are no single nanoparticles available anymore. Similar features have been reported in literature.^6^

The boundaries between the LD and HD domains are sharp, typically 25 nm wide, which comprises less than one string of typical length of 50 to 100 nm. The shape of the boundary is determined by kinetics and the energy of the boundary (which is an energy per unit length in this 2D case). No tendency of LD-HD interface faceting is observed, which indidates that the boundary energy is low and isotropic. In a number of cases, the segregation of the LD and HD domains has not yet finished as shown in Figure 6. This reflects an early state of monolayer growth, characterized by a mixture of strings and isolated nanocrystals, which subsequently segregate into the two distinct phases.

As mentioned above, the empty regions in Figure 3 are formed by rupture of the monolayer film during separation from the EG substrate prior to TEM observation. Only the TEM background carbon film is imaged. Interestingly, the ruptures traverse the LD and HD domains showing that these two domains are formed prior to separation, as visible in Figure 7. In some cases a fracture can be revealed within a single LD or HD domain and the fracture patterns of two adjacent patches often show an opposite shape. The fracture edges of the LD and HD domains are sharp and show no evidence of rounding by reorganization, which indicates that these domains behave solid-like or as a liquid of high viscosity.

**Figure 7:**
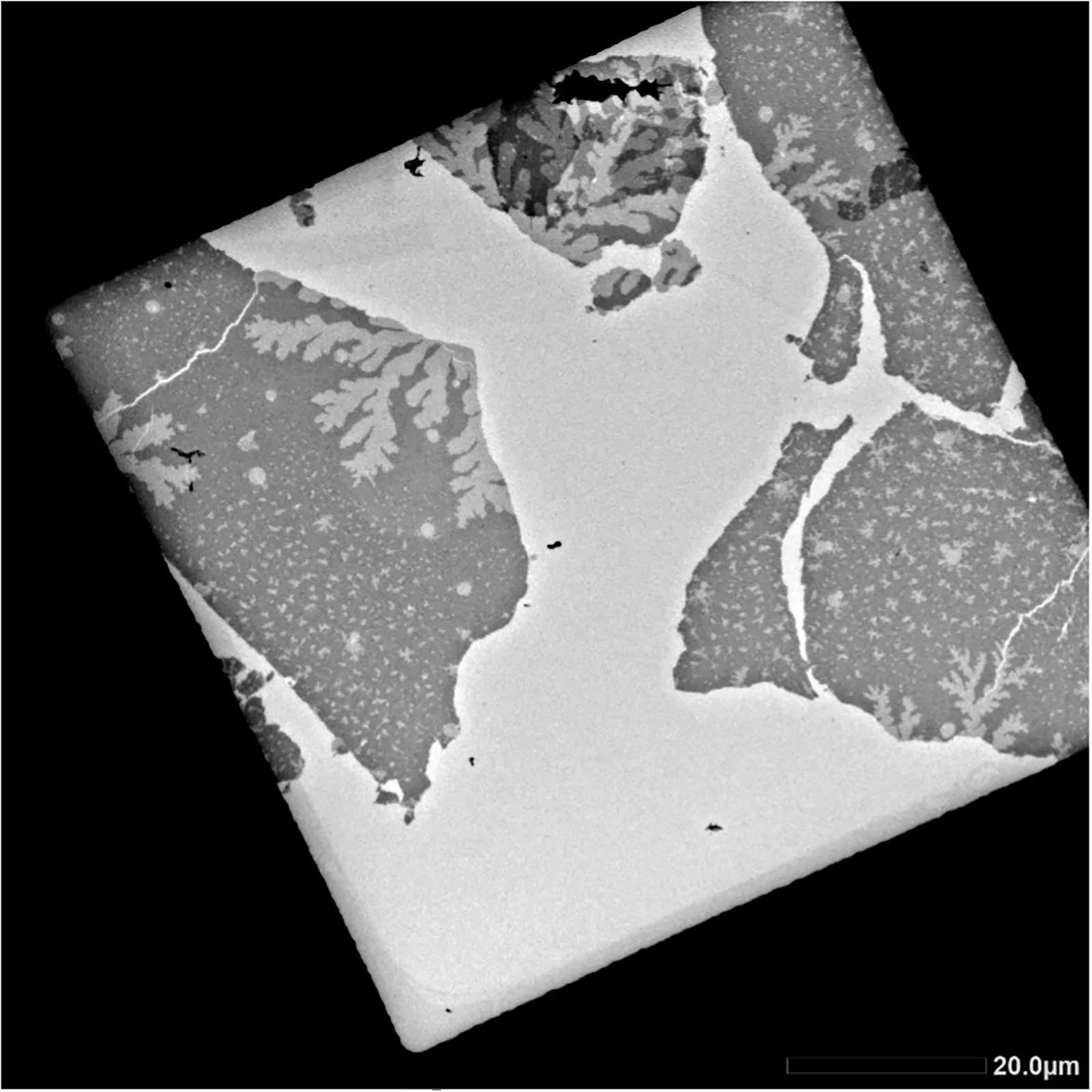
Overview of a sample where a continuous monolayer was broken in multiple pieces.

### 3.2 Concentration Effects and Pattern Formation

We have performed experiments where we have kept the experimental protocol mostly unchanged, except for the concentration. We performed experiments with 4 to 9 μL of nanocrystal solution. Figure 8 gives an overview of the formation of the three different monolayer types as a function of nanocrystal surface concentration as found during our experiments. As pointed out before, 6 μL stock solution corresponds with one monolayer of nonattached quantum dots.

**Figure 8:**
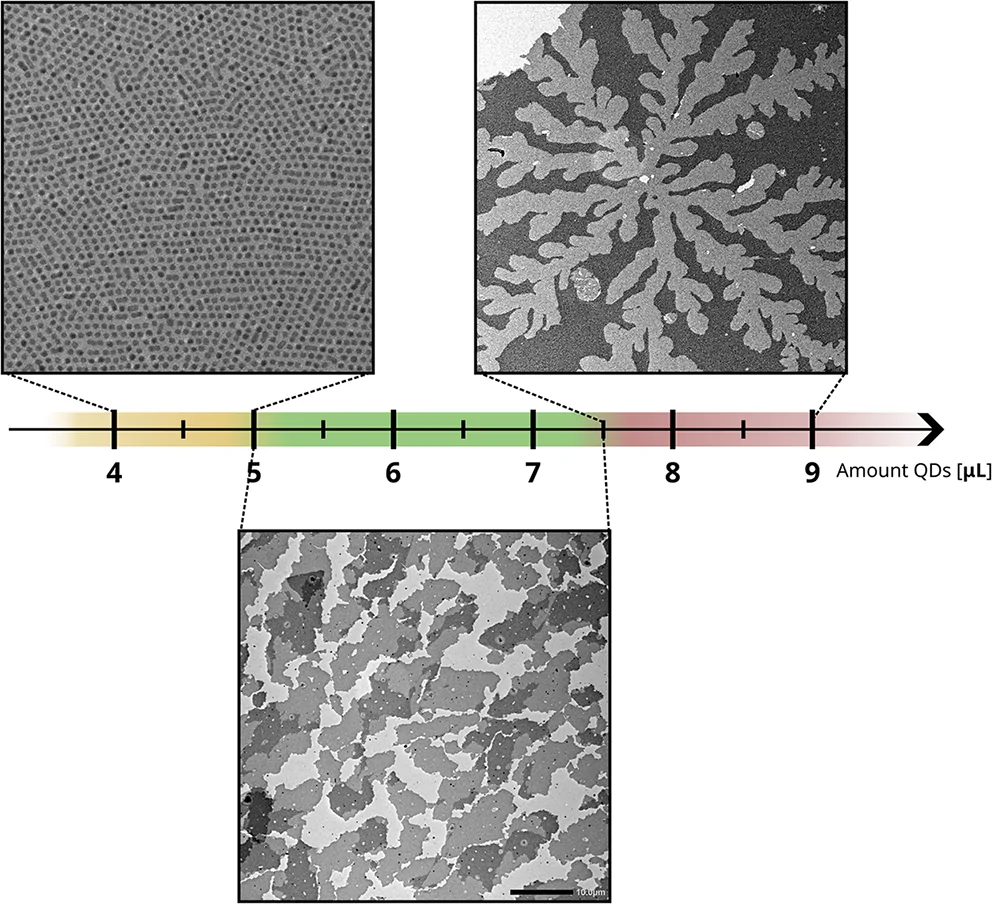
TEM images showing three different regimes found in a monolayer PbSe nanocrystal film obtained after evaporation of a suspension in 35 μL octane acquired from 4 μL, 7 and 9 μL stock solution. The different highlighted ranges represent the regimes in which those particular morphologies were found: in yellow samples with only LD regions, in green where regions were equally mixed between LD and HD and in red densities where dendrites have been found.

The experiments using the 4 μL stock solution showed only LD domains; HD domains were not observed. This indicates that attachment of the nanocrystals does not take place. Here the surface coverage is less than a monolayer and the 2D pressure in the layer is low. This suggests that lateral compression of the monolayer is essential for inducing nanocrystal attachment in our system. Increasing the layer pressure by adding more (5 to 7.5 μL) stock solution to the system leads to the formation of separate HD and LD domains and intermediate LD/HD domains (Figure 6).

For the 7.5 to 9 μL stock solution cases dendritic LD growth patterns emerge in extended HD layers as shown in Figures 1 and 9. The dendrites show no preferential orientation and thus are seaweed type.^16^ In contrast to dendrites obtained by classical Langmuir monolayer experiments of polar molecules on water substrates,^17,18^ in our case the dendrites are made up of the low density rather than the high density phase. Again the boundary between the two domains is sharp (Figure 9b). The branched LD dendrite patterns emerge from one single point in the HD layer. The density of surrounding small LD nuclei decreases for decreasing distances from the dendrite arms (Figures 1a, 7, and 9b), which indicates depletion of nonattached nanocrystals in the inner dendrite regions. In addition, near the center of the dendrites the patterns are more smooth than at their periphery (Figure 9a). This is explained by a lowering of the total edge free energy in time, which is the early stage of two-dimensional Oswald ripening. This points to a reversible interconversion of the HD ↔LD domains. As dendrites are the product of diffusion-controlled growth,^19,20^ this indicates that nonattached nanocrystals are mobile within the HD layer.

**Figure 9:**
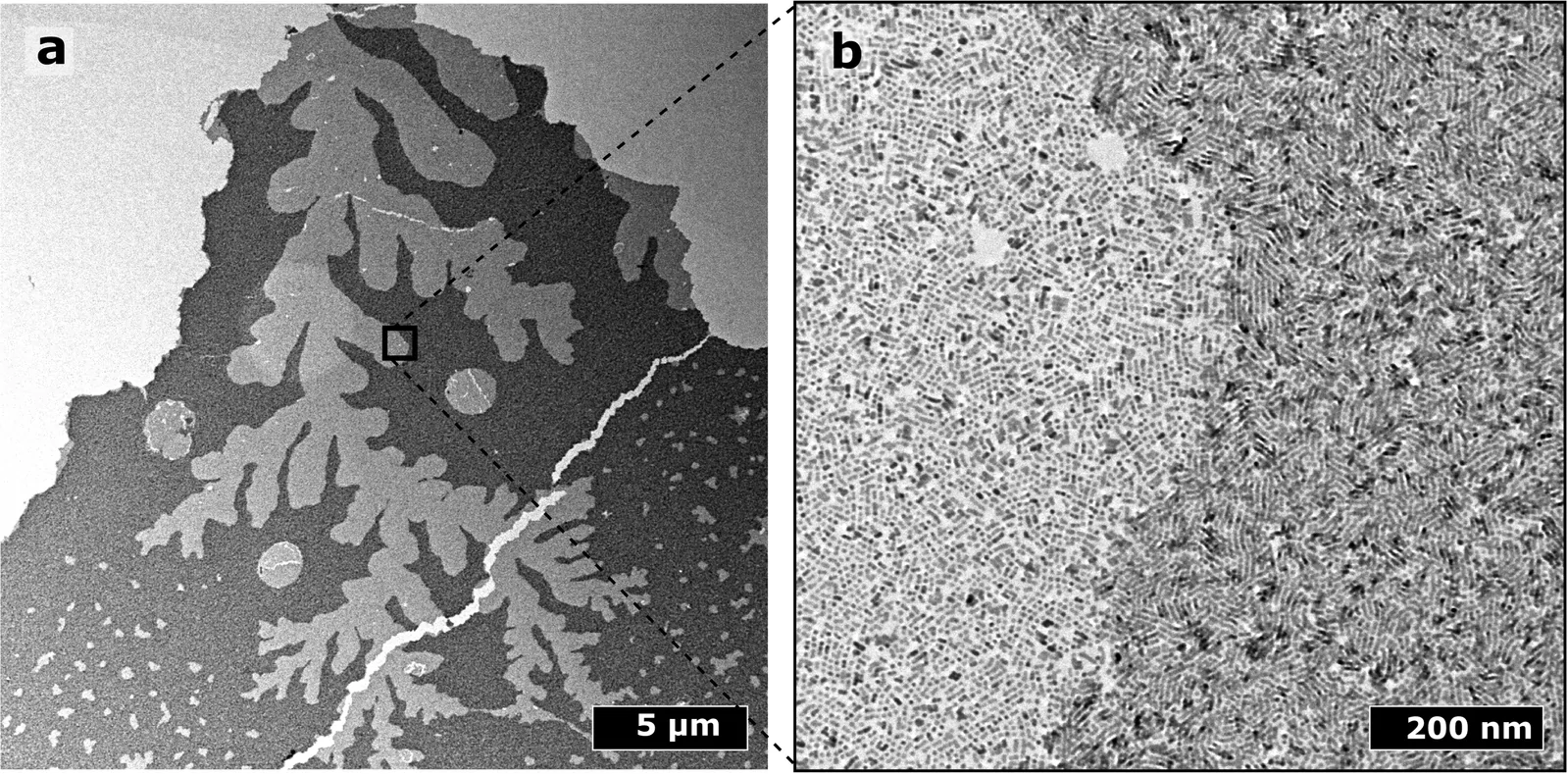
TEM images of dendritic growth patterns. (a) is a representative region. (b) zoomed in area from a sample, highlighting the microstructure of the dendrites found at much smaller scales.

## 4 Discussion

Two distinct phases (LD and HD) have been identified, and the experiments have shown a clear dependence of the system on the concentration of the nanoparticles at the surface. The driving force of these alternations is surface pressure, imposed by steric effects, which is directly related to the surface concentration of the nanoparticles.

For the lowest concentrations (less than 5 μL stock solution) where the EG substrate is only partly covered, the nanoparticles do not undergo sufficient surface pressure to initiate attachment and only LD patterns form. Here the nanocrystals remain fully covered by ligands and only interact by weak van der Waals forces forming a continuous closed packed LD layer (Figure 4).

For intermediate amounts of stock solution from 5 μL up to 7.5 μL, the surface coverage approaches or exceeds monolayer coverage and HD domains are formed as well (Figure 3). This is the result of a rise in surface pressure by which the activation barrier for attachment is overcome due to forced interaction between the units. This leads to the development of attached nanocrystal strings, which then rearrange by mutual, parallel alignment into HD domains in a LD “solvent”. The formation of the HD domains from the originally nonattached nanocrystals leads to a shrinkage of the layer, making room for LD domains. This is a consequence of the higher density of the HD layers as compared to the LD layers, 2.70 × 10^4^ versus 1.25 × 10^4^ nanocrystals/μm^2^, which gives a contraction of ≈50% in surface area. The process of HD growth from LD gradually lowers the overall pressure and continues until dynamic equilibrium between the two phases is obtained. From thermodynamics it is well-known that a higher pressure promotes the development of a higher density phase.

Using higher concentrations, starting from 7.5 μL up to 9 μL stock solution, dendrite patterns composed of LD layers embedded in a HD matrix are formed. For these concentrations, the surface coverage largely exceeds monolayer coverage of nonattached nanocrystals, which makes surface pressure high. This induces an initial, rapid formation of numerous attached nanocrystal strings resulting in an extended HD layer studded with nonattached nanocrystals in its vacant sites (Figure 6). This is a consequence of the fact that the nonattached nanocrystals do not adhere to the sides of the nanocrystal strings, so that many nonattached units remain in the network of rapidly formed strings. In this way a supersaturated solution of nonattached units in the HD layer is formed. Then, this layer shrinks by diffusion transport of the nonattached units out of the HD layer. In this way it makes room for and generates LD phase domains and the dendrite patterns. It is important to note that the driving force of this process is the contraction of HD layers by alignment of the chains of attached nanocrystals. This leads to expulsion of nonbonded nanocrystals from the HD layer, which lowers surface pressure and creates low pressure LD regions. The observed smoothing of the patterns near the dendrite centers and the rounded shapes of the patches between the dendrite arms (Figure 9a) are typical for a late stage of dendrite growth where the driving force is very low. The rounded shapes are likely the result of a final, slow reordering of the nanocrystal chains minimizing the total, isotropic, LD-HD boundary length and therefore energy. The diffusion transport of the nonattached nanocrystals likely proceeds via voids between the attached chains in the HD layers. In addition, one can also speculate on transport over the HD layer or through the upper EG substrate layer underneath the HD layer, but we have no experimental data supporting or refuting that.

In our system at high nanocrystal concentration the conditions for dendrite formation are met.^15,16^ First, the driving force, i.e., surface pressure, is high. Second, we have two separate phases in which LD forms from a HD solution. The interdomain line energy between both phases is low as the periphery of the strings as well as the nonbonded nanocrystals are covered by ligands and only interact by the weak van der Waals forces. Third, the process of LD domain/dendrite growth from the HD layer is controlled by diffusion-limited transport of nonbonded units toward the LD/HD interface.

The LD dendrites have a ≈50% lower nanocrystal surface density than the surrounding HD medium. Formation of low density dendrites from a high density phase is unusual. Normally, in dendrite formation mass transport goes from low density to high density phases. In our case the HD regions at their earliest stage of formation can be seen as a supersaturated solution of LD monomers. Due to further rearrangement of the linear chains in the HD phase, isolated LD units are expelled, locally lowering energy but increasing entropy. The formation of the LD domains clearly lowers the overall free energy, giving an effective thermodynamic driving force for LD domain growth and HD domain contraction. This is opposite to the case of intermediate nanocrystal concentrations, where the LD phase acts as a solvent for nanocrystal strings rearranging into HD domains.

In our work instead of the commonly used toluene, a different carrier solvent, octane, was used. It is likely that the properties of octane are required for the observed dendritic growth. This solvent is less prone to dissolve and remove the oleic acid ligands from the quantum dot surfaces as compared to toluene. So, here the activation barrier for ligand removal is higher, reducing the chances for attachment. It is known that the reactivity of individual nanoparticles is correlated with their average coverage of ligands. Toluene can solvate more ligands: this might lead to a situation where, under the experimental conditions used here, nanocrystals are all used up in the oriented attachment process. In that case a completely attached monolayer is formed, preventing the formation of dendrites in the samples.

## 5 Conclusions

PbSe nanocrystal monolayers have been deposited on ethylene glycol substrates as a function of nanocrystal concentration in the octane solvent. In several of these monolayers remarkable 2D dendritic patterns were encountered, which needed explanation. Two domains were distinguished: low and high density domains, denoted respectively by LD and HD. The former is composed of a close packing of nonattached nanocrystals covered by oleate ligands, while the latter is composed of strings of attached nanocrystals. For the lowest nanocrystal concentrations, i.e., lowest surface pressures, only LD domains form. Intermediate concentrations -i.e., surface pressure- produce both LD and HD monolayer areas. Here, the original LD layer -partially- transforms into higher density HD layers, leaving space for LD domains. For the highest nanocrystal concentrations LD seaweed dendrite patterns develop by diffusion controlled expulsion of nonattached nanocrystals from extended rapidly formed HD layers packed with these units. The driving force for this process is shrinkage of the HD layers induced by further attachment of nonbonded nanocrystals as well as alignment of the attached nanocrystal chains, making room for LD dendrite formation, lowering interaction free energies and entropy. It is interesting to note that at intermediate concentrations HD domains grow from a LD phase, which is the opposite of LD dendrite growth from a HD phase at high concentrations.

## Supplementary Material



## Notes

Bilayer model as alternative explanation of string observation (PDF)
