# Supplementary material for: Domain and Dendrite Pattern Formation in PbSe Nanocrystal Monolayers
Source: Cryst Growth Des. 2026 Aug 3;26(16):6399–406. doi: 10.1021/acs.cgd.6c00339 (PMC13495671; doi:10.1021/acs.cgd.6c00339)
Supplement: Supplementary file 1 [file cg6c00339_si_001.pdf]

Domain and dendrite pattern formation in PbSe nanocrystal monolayers

Paolo Accordini, Willem J.P. van Enckevort and Elias Vlieg*

Radboud University, Institute for Molecules and Materials,
Heyendaalseweg 135, 6525 AJ, Nijmegen, The Netherlands

1 Bilayer model for string formation

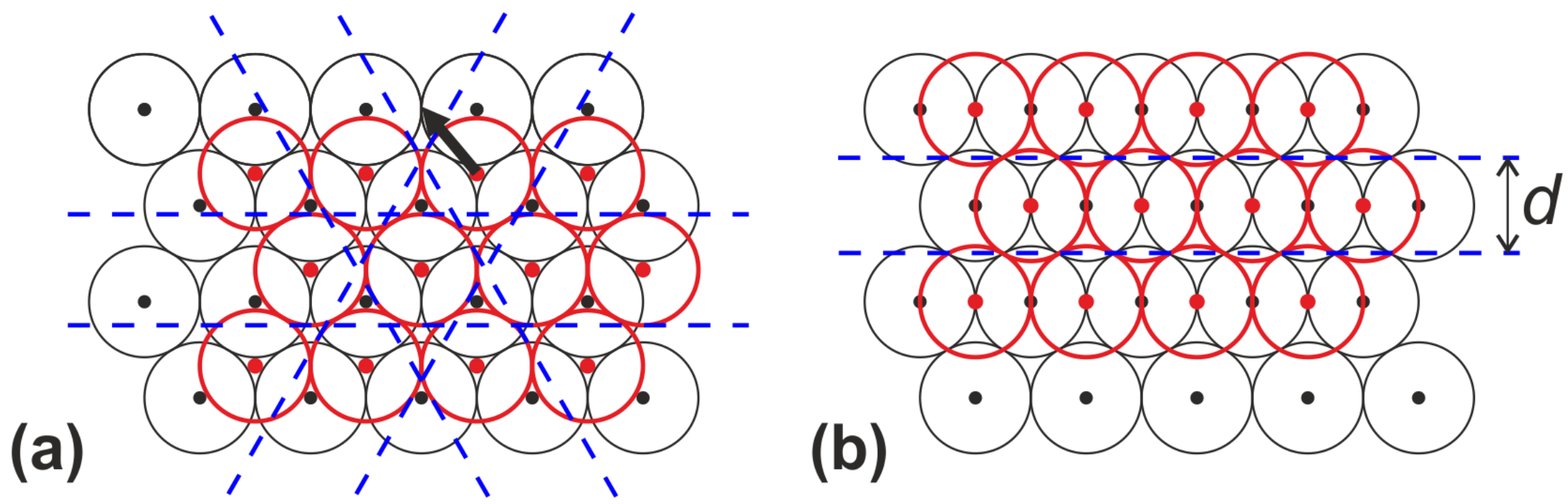

Fig. S1: Schematic view of the hypothetical two layer HD structures: bottom (LD) layer in black, top layer in red, suggested string directions in dashed blue. The solid dots are the projections of the PbSe cores of the non-attached nanocrystals (for clarity, size not to scale). (a) Lowest energy configuration, not revealing an unidirectional string pattern; (b) Unidirectional strings can be produced by shifting the top layer over a distance indicated by the arrow. Here the expected string separation ($d = \sqrt{3}/2$ of the diameter unattached nanocrystal) is close to the observed value. However, as each upper layer sphere contacts only two spheres of the layer below, this structure is energetically unfavourable and therefore unlikely.

An alternative explanation for the observed low-density (LD) and high-density (HD) layers is that the HD layer consists of close-packed, unattached nanocrystals positioned above a close-packed LD layer, as schematically shown in Figure S1a. Because TEM images are projections, this stacked arrangement could produce the darker contrast observed for the HD layer. At lowest concentrations only one layer forms, at intermediate concentrations HD islands develop on top of the LD layer and at the highest concentration a high density top layer forms filled

with numerous vacancies. After some time the vacancies diffuse out of the layer accumulating into the dendrite patterns, which are vacant areas on top of the LD layer. However, this hypothesis does not explain the formation of the unidirectional string patterns as observed by TEM. In this case one would expect a more or less uniform or trigonal symmetric pattern. The formation of linear strings requires a large change of the structure. The most favourable way to do this is by shifting the top layer over the LD layer as indicated by the arrow in figure S1a, which results in a pattern with unidirectional strings as shown in figure S1b. Here, each top layer sphere contacts only two spheres below instead of three as is the case for the lowest energy configuration in figure S1a. This is highly unfavourable from an energy point of view and not likely to occur. In addition, isolated strings not related to a LD layer were commonly observed as well (figure S2). So, no sublayer is needed for string formation.

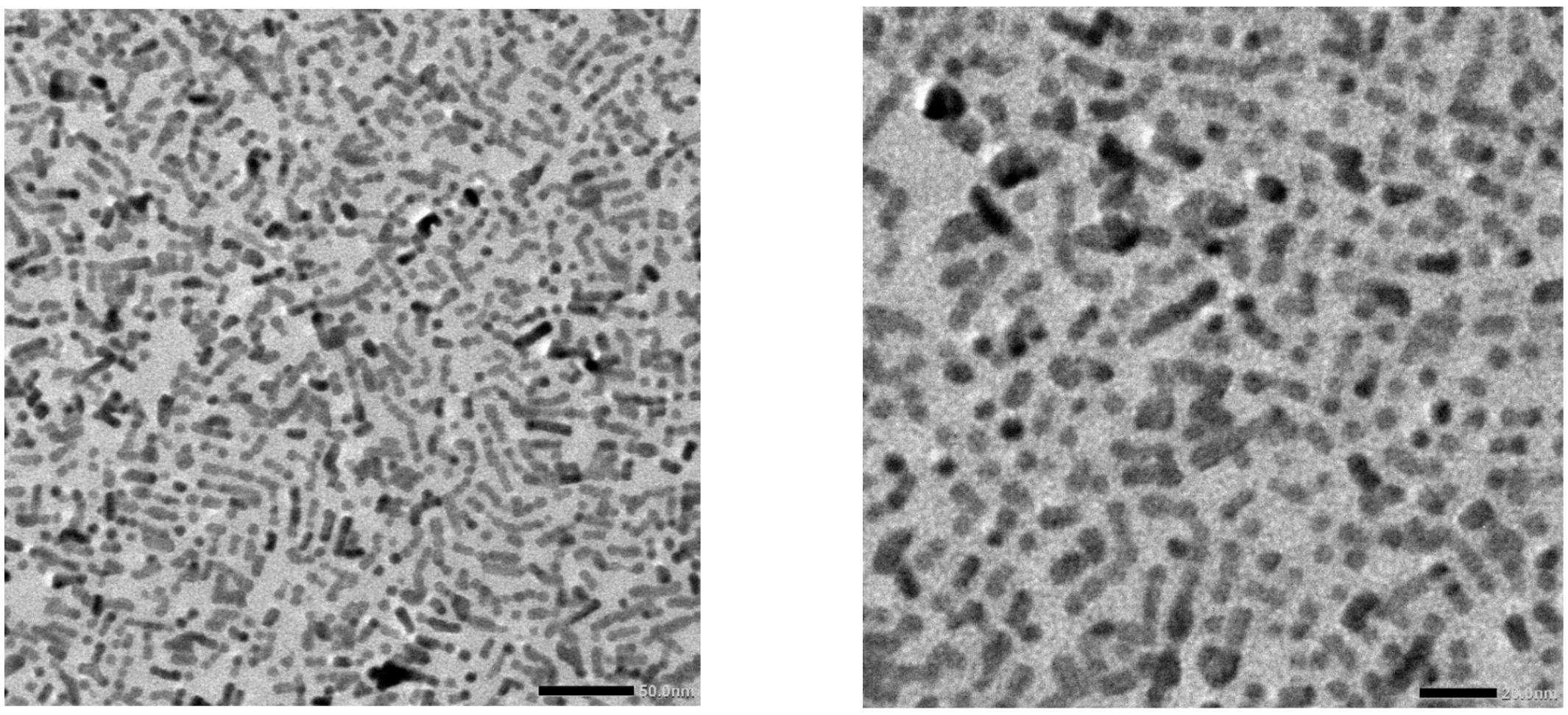

Fig. S2: TEM micrographs of isolated nanocrystal strings not related to a LD layer. These patterns appear in the same samples that contain the domain and dendrite patterns.
